# Return Encounters in Emergency Department Patients Treated with Phenobarbital Versus Benzodiazepines for Alcohol Withdrawal

**DOI:** 10.1007/s13181-021-00863-2

**Published:** 2021-10-25

**Authors:** Jacob A. Lebin, Anita Mudan, Charles E. Murphy, Ralph C. Wang, Craig G. Smollin

**Affiliations:** Department of Emergency Medicine, University of California, San Francisco, 1001 Potrero Ave, Building 5, Room 2C8, Box 1369, San Francisco, CA 94143 USA

**Keywords:** Phenobarbital, Benzodiazepines, Alcohol withdrawal, ED return visit

## Abstract

**Introduction:**

Phenobarbital has been successfully used in the emergency department (ED) to manage symptoms of alcohol withdrawal, but few studies have reported outcomes for ED patients who receive phenobarbital and are discharged. We compared return encounter rates in discharged ED patients with alcohol withdrawal who were treated with benzodiazepines and phenobarbital.

**Methods:**

This is a retrospective cohort study conducted at a single academic medical center utilizing chart review of discharged ED patients with alcohol withdrawal from July 1, 2016, to June 30, 2019. Patients were stratified according to ED management with benzodiazepines, phenobarbital, or a combination of both agents. The primary outcome was return ED encounter within three days of the index ED encounter. Multivariate logistic regression identified significant covariates of an ED return encounter.

**Results:**

Of 470 patients who were discharged with the diagnosis of alcohol withdrawal, 235 were treated with benzodiazepines, 133 with phenobarbital, and 102 with a combination of both. Baseline characteristics were similar among the groups. However, patients who received phenobarbital were provided significantly more lorazepam equivalents compared to patients who received benzodiazepines alone. Treatment with phenobarbital, alone or in combination with benzodiazepines, was associated with significantly lower odds of a return ED visit within three days compared with benzodiazepines alone [AOR 0.45 (95% CI 0.23, 0.88) *p* = 0.02 and AOR 0.33 (95% CI 0.15, 0.74) *p* = 0.007].

**Conclusions:**

Patients who received phenobarbital for alcohol withdrawal were less likely to return to the ED within three days of the index encounter. Despite similar baseline characteristics, patients who received phenobarbital, with or without benzodiazepines, were provided greater lorazepam equivalents the ED.

**Supplementary Information:**

The online version contains supplementary material available at 10.1007/s13181-021-00863-2.

## Introduction

### Background

Alcohol withdrawal is a common, potentially life-threatening complication of chronic alcohol use and is associated with significant emergency department (ED) utilization [1]. More than eight million people in the USA meet the criteria for an alcohol use disorder (AUD), and nearly half of these individuals will experience some symptoms of alcohol withdrawal when alcohol intake is either reduced or discontinued [2, 3]. For patients with moderate to severe alcohol withdrawal symptoms, benzodiazepines are considered the mainstay of treatment. However, benzodiazepines often require frequent redosing, and prolonged use has been associated with delirium [4].

During the national intravenous diazepam shortage in 2017, phenobarbital emerged as an attractive alternative due to its predictable loading pharmacokinetics and long duration of action [5]. In fact, previous studies have shown phenobarbital, with or without benzodiazepines, to be associated with decreased rates of ICU admission and shorter hospital length of stay without an increased risk of adverse events [6, 7]. However, most ED-based studies utilizing phenobarbital have largely focused on inpatient metrics despite the risk for respiratory depression if phenobarbital is combined with alcohol or other sedatives in discharged ED patients [6–9]. Only one study, a single prospective, randomized trial of phenobarbital versus lorazepam, has specifically examined the outcomes of alcohol withdrawal patients who are discharged from the ED, but was significantly limited by the number of patients lost to follow-up [10].

We hypothesized that the rate of ED return visits would be lower in discharged ED patients with alcohol withdrawal who received phenobarbital, alone or in combination with benzodiazepines, when compared to patients who received benzodiazepines alone.

## Methods

### Study Design and Setting

This was a retrospective cohort study at a single, urban academic medical center with an annual census of 44,000 emergency department visits. At this institution, physicians have wide latitude in choosing the diagnostic testing, treatment, and disposition for patients presenting with alcohol withdrawal. There are no departmental assessment or treatment guidelines, and the Clinical Instrument Withdrawal Assessment-Alcohol Revised (CIWA) is not routinely utilized to guide management. Institutional review board approval was obtained for all study procedures. This study was designed and prepared according to STROBE guidelines [11].

### Selection of Participants

We collected data for consecutive, adult patients from July 1, 2016, to June 30, 2019, who were discharged from the ED with a discharge diagnosis containing the keyword “alcohol withdrawal.” Physicians are required to list at least one discharge diagnosis per encounter. We excluded patients who were incarcerated, transferred, or discharged to another facility because interactions with a receiving facility may have influenced the observed outcomes. We also excluded patients who did not receive pharmacologic treatment for alcohol withdrawal with either a benzodiazepine or phenobarbital, which suggested either a very mild presentation or an alternative diagnosis.

### Data Collection and Processing

Data were abstracted from the hospital-based electronic medical records (EMR) with a point-to-point health information exchange embedded in the EMR. Our institution uses the Epic Hyperspace EMR and health information exchange Care Everywhere (Epic Systems Corporation, Verona, WI). Briefly, Care Everywhere is an electronic platform that facilitates the sharing of patient health information across disparate healthcare organizations who are involved in the treatment of the same patient. Demographics, triage vital signs, and administered medications were abstracted from the hospital EMR. The co-variates of history of liver disease, history of substance abuse disorder, and history of delirium tremens were also abstracted from the hospital EMR and identified by ICD code (available in supplement). Patients with a history of alcohol withdrawal seizure were included in the delirium tremens co-variate as it was felt that this represented more severe AUD.

The presence of a return ED encounter was manually queried and abstracted by two authors (JAL and AM) using Care Everywhere. In our system, Care Everywhere can query the medical records of twelve regional health organizations for any patient, representing approximately 75% all regional EDs. Return visit diagnosis and disposition were also manually abstracted from ED documentation available from any regional healthcare organization in Care Everywhere. All data abstracted by manual review were confirmed by both reviewers. Full consensus between reviewers was required for inclusion. If consensus was not reached, the case was adjudicated by a third reviewer.

### Outcome Measures

The primary outcome of the study was a return ED encounter within three days of the index ED encounter. A return encounter was defined as any ED patient encounter, at any regional institution, following the index ED encounter. Return encounters were further categorized as either occurring within three or between three and seven days following the index visit, as the effect duration of phenobarbital is approximately three days [5]. Secondary outcomes included diagnosis and disposition for the return encounter. In an effort to evaluate the presence of missed return ED encounters due to a patient death, short-term survival following the index ED encounter was surveyed. This outcome was exploratory, and we were not powered to detect small, but potentially clinically meaningful, differences in survival. Short-term survival was defined as any healthcare encounter, either at our institution or any regional institution, documenting that the patient was alive beyond three days of the index ED visit. Documentation of short-term patient survival was manually queried and abstracted by two authors (JAL and AM) using Care Everywhere.

### Exposure of Interest

Choice of medication, dosing, and routes of administration were at the discretion of the treating physician and abstracted from the EMR’s Medication Administration Record. Phenobarbital was available in both intravenous (IV) and intramuscular (IM) formulations. The following benzodiazepines were available in our ED: lorazepam (IV, IM, and oral), diazepam (IV, IM, and oral), midazolam (IV), and chlordiazepoxide (oral). Any dose of a given medication was considered an exposure for the purpose of analysis.

### Analysis

**Fig. 1:**
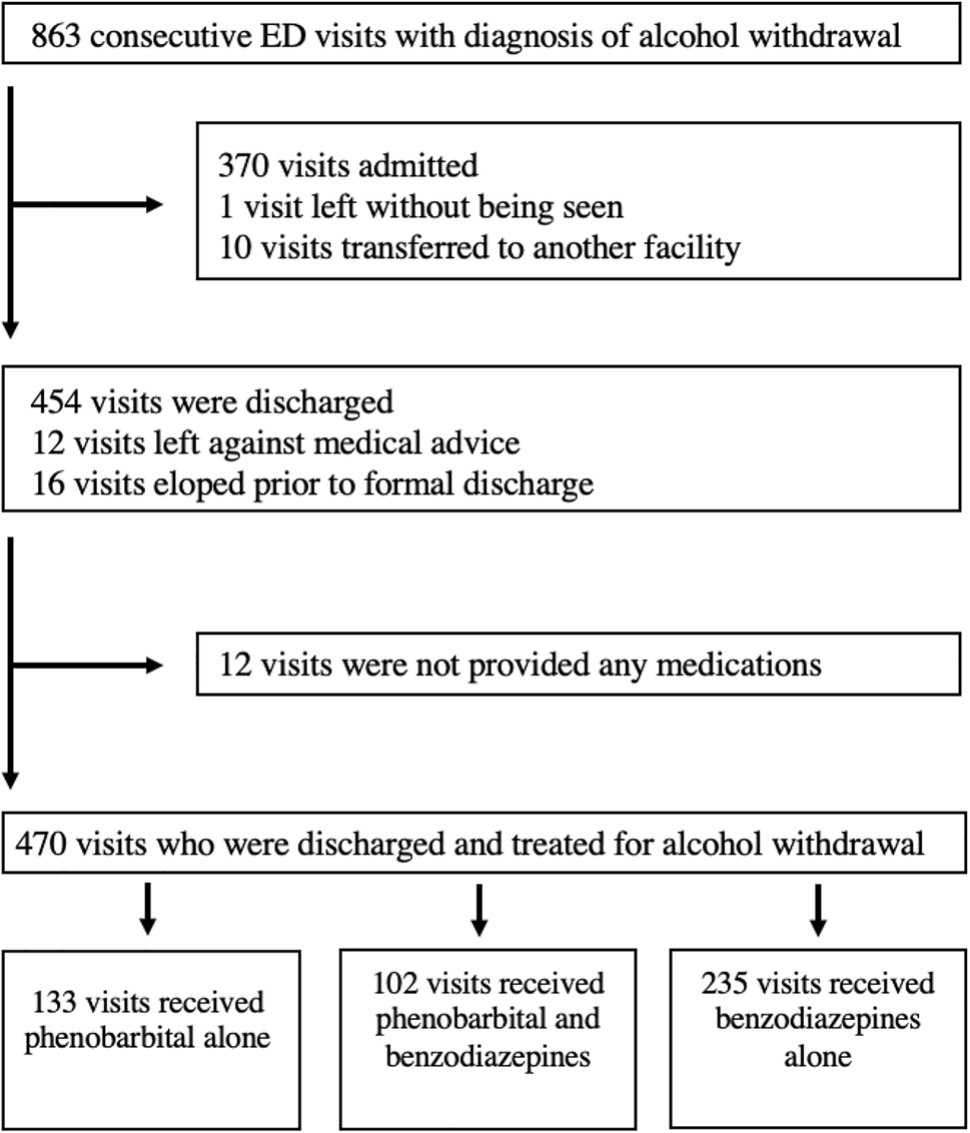
Study flow diagram

We classified ED visits into three treatment categories: benzodiazepines, phenobarbital, and combination therapy with both phenobarbital and benzodiazepines (Fig. 1). We identified a comorbid substance use disorder and age as possible confounders. As we did not have access to CIWA scores, we included a past history of delirium tremens or liver disease as surrogate markers for the severity of alcohol use. These covariates, along with age and a history of substance use disorder, were included in the multivariate analyses of the primary outcome.

Using an assumption of a medium effect size (f = 0.39), eight predictors, desired power of 0.8 and alpha 0.05, we calculated a minimum sample size of 107 visits. Normality of continuous data was assessed by visual inspection of frequency distribution plots and Shapiro–Wilk test. When data were not normal (phenobarbital and benzodiazepine dose), Kruskal–Wallis test was used to assess differences across treatment categories. When data were normal, one-way ANOVA was used to evaluate the association of categorical variables with continuous outcomes. In all analyses, we used the Wald test to evaluate the main effect of treatment category in the final models and to test for heterogeneity across the three categories. *Z* tests were used to evaluate differences between the treatment categories in the final models. Significance level was set at 0.05.

As patients may present to the ED more than once, repeated measures on individual patients are possible. To address the impact of clustering within patients, we used mixed effects logistic regression for the analyses of return ED visits. For all analyses, we used individual patient clusters indicated by their medical record numbers. All results displayed and discussed here are visit level, not patient level. We checked our results by conducting a sensitivity analysis of the final models including robust standard error estimations. All models were adjusted for age, history of delirium tremens, history of substance use disorder, and history of liver disease. Wald’s Chi-squared test was used to assess overall model performance, and the likelihood ratio test was used to compare the results of the mixed effects logistic models. Prior to modeling the data, we conducted diagnostic checks to evaluate for influential points and multiple collinearity using box plots of df_beta, variance inflation factor, and tolerance. We found no evidence of multiple collinearity or influential points. All statistical analyses were conducted using STATA MP v15 (STATA corp, College Station, TX).

## Results

During the study period, there were 470 individual discharged ED visits related to alcohol withdrawal, representing 285 unique patients. Benzodiazepines were used in 235 visits, phenobarbital in 133 visits, and a combination of both agents in 102 visits. The kappa score for inter-reviewer agreement regarding the presence of a return ED encounter within three days was 0.92.

The characteristics of ED visits for each treatment group are displayed in Table 1. We found no significant differences in the prevalence of liver disease, substance use disorders, or delirium tremens between the groups. Mean age, triage heart rate, and triage blood pressure were similar across treatment categories. Race varied significantly across visit categories. Visits where phenobarbital was used alone had a lower proportion of white/Caucasians compared with other groups.

**Table 1:** Baseline variables of patients discharged for alcohol withdrawal by treatment group

Variable	Benzodiazepine only (*N* = 235)	Phenobarbital only (*N* = 133)	Combination (*N* = 102)	*p* Value
Demographics and history				
Men	189 (80)	112 (85)	85 (83)	0.5
White	187 (80)	94 (71)	84 (82)	0.05
Age median (IQR)	44 (12)	45 (11)	45 (11)	0.4
History of delirium tremens	63 (27)	50 (38)	31 (31)	0.1
History of substance use disorder*	11 (5)	8 (6)	7 (7)	0.6
History of liver disease**	13 (6)	6 (5)	1 (1)	0.16
Initial ED vital signs, mean (sd)				
Heart rate, beats/min	101 (16)	101 (17)	103 (17)	0.45
Systolic blood pressure, mm Hg	139 (23)	138 (18)	148 (19)	0.24
Diastolic blood pressure, mm Hg	84 (14)	85 (12)	87 (12)	0.15
ED management, median total dose, mg (IQR)				
Phenobarbital	0	390 (260, 520)	357.5 (260, 520)	0.03
Benzodiazepine in mg of lorazepam	6 (3.5, 9)	0	4 (2, 5)	< 0.001
Chlordiazepoxide	165 (70)	0	43 (42)	< 0.001
Benzodiazepine equivalents in mg of lorazepam	6 (3.5, 9)	26 (17.3, 34.7)	28 (20.3, 36.7)	< 0.001
Outcome				
Survival	222 (94.5)	130 (97.7)	94 (92.2)	0.12

Data are presented as No. (%) unless otherwise indicated

^*^Substance use disorder: opioid abuse, opioid dependence, inhalant abuse, psychoactive substance use, sedative/hypnotic dependence, stimulant dependence

^**^Liver disease: esophageal varices, alcoholic cirrhosis, alcoholic fatty liver, alcoholic hepatitis

The median dose of phenobarbital was larger in the phenobarbital only group compared to the combination phenobarbital and benzodiazepine group (390 mg vs 357.5 mg, *p* = 0.03). The median dose of benzodiazepines was significantly greater in the benzodiazepine only group compared with the combination phenobarbital and benzodiazepine group (equivalent to 6 mg vs 4 mg lorazepam, *p* < 0.001). When expressed in lorazepam equivalents, the benzodiazepine group received significantly less total lorazepam equivalents than either the phenobarbital only or combination phenobarbital and benzodiazepine group (*p* < 0.001) [13]. The frequencies of various combinations of drugs used are available as a supplement. Briefly, chlordiazepoxide and lorazepam were the most frequently used benzodiazepines, and the two were often combined with each other or with phenobarbital. Diazepam alone or in combination with other benzodiazepines was also commonly used but was rarely combined with phenobarbital. Midazolam use was rare.

### Main Results

The results of the bivariate analysis are displayed in Table 2. Treatment group was a significant predictor of a repeat ED visit within three days, but not between three and seven days. Twenty-five percent of the benzodiazepine group had a return visit within three days compared to 10% of the phenobarbital monotherapy and 13% of the combination benzodiazepine and phenobarbital group (*p* = 0.001). Ten percent of the benzodiazepine group had a return visit between three and seven days after the index visit, compared with 6% of the phenobarbital mono-therapy group and 11% of the combination benzodiazepine and phenobarbital (*p* = 0.36).

**Table 2:** Bivariate analysis of association of alcohol withdrawal treatment and return ED visit

Variable	Benzodiazepine only (*N* = 235)	Phenobarbital only (*N* = 133)	Combination (*N* = 102)	*p* Value
ED visit within 3 days	58 (25)	17 (13)	10 (10)	0.001
ED visit within 7 days	76 (32)	24 (18)	20 (20)	0.003
ED visit within 3–7 days	18 (10)	7 (6)	10 (11)	0.36

Data are presented as No. (%) unless otherwise indicated

The measures of association [unadjusted odds ratio (OR) and adjusted odds ratios (AOR)] from the mixed effects models are displayed in Table 3. In the unadjusted model, treatment group remained a significant predictor of a return ED visit within three days, but not between three and seven days (*p* = 0.01 and 0.5 respectively). Treatment with phenobarbital, alone or in combination with benzodiazepines, was associated with significantly lower odds of a return ED visit within three days compared with benzodiazepines alone [unadjusted OR (95% CI), 0.49 (0.25, 0.96) and 0.35 (0.16, 0.77), respectively]. When comparing the group treated with phenobarbital plus benzodiazepines to the group treated with phenobarbital alone, no statistically significant difference in the odds of a return ED visit within three days was found [unadjusted OR 0.71 (0.28, 1.8)]. After adjustment, treatment group remained a significant predictor for a return ED visit within three days, but not between three and seven days (*p* = 0.006 and 0.47 respectively). Treatment with phenobarbital, alone or in combination with benzodiazepines, was associated with significantly lower odds of a return ED visit within three days compared with benzodiazepines alone [AOR 0.45 (95% CI 0.23, 0.88) *p* = 0.02 and AOR 0.33 (95% CI 0.15, 0.74) *p* = 0.007, respectively]. When comparing treatment with phenobarbital alone to phenobarbital plus benzodiazepines, no statistically significant difference was apparent in the odds of a return ED visit within three days was found [AOR 1.7 (0.6, 5.2 *p* = 0.3)].

**Table 3:** Adjusted measures of association between alcohol withdrawal treatment and return ED visit

Variable	Benzodiazepines only (*N* = 235)	Phenobarbital only (*N* = 133)	Combination (*N* = 102)	*p* Value (PB vs BZD, PB + BZD vs BZD)
Unadjusted mixed effects model (OR, 95% CI)				
ED visit within 3 days	Ref	0.49 (0.25, 0.96)	0.35 ( 0.16, 0.77)	0.04, 0.009
ED visit within 7 days	Ref	0.51(0.26,0.99)	0.53 (0.25, 1.1)	0.048, 0.086
ED visit within 3–7 days	Ref	0.59 (0.22, 1.6)	1.06 (0.44, 3.1)	0.3, 0.9
Adjusted mixed effects model (aOR, 95% CI)				
ED visit within 3 days	Ref	0.45 (0.23, 0.88)	0.33(0.15, 0.74)	0.02, 0.007
ED visit within 7 days	Ref	0.45 (0.23, 0.88)	0.50 (0.26, 0.99)	0.02, 0.047
ED visit within 3–7 days	Ref	0.55 (0.2, 1.5)	0.96 (0.37, 2.5)	0.25, 0.9

There were 120 ED return visits of which 92 were associated with a diagnosis of alcohol withdrawal or intoxication. Thirteen of the return ED visits resulted in admission to the hospital. Eleven of these admissions had a diagnosis of alcohol withdrawal or intoxication. There were no significant differences between treatment groups in the proportion of return visits resulting in admission or the proportion of return visits associated with an alcohol-related diagnosis (exact *p* = 0.7 and 0.2 respectively). We were unable to confirm survival (i.e., lost to follow-up) following the index encounter in 24 cases with no apparent differences between treatment groups.

### Sensitivity Analysis

Our results were similar when comparing phenobarbital alone to treatment with chlordiazepoxide (with or without other benzodiazepines). Phenobarbital alone was associated with significant lower odds of a return ED visit within three days [AOR 0.48 (95% CI 0.23, 0.99) *p* = 0.049], but not between three and seven days [AOR 0.78 (95% CI 0.22, 2.7) *p* = 0.8].

## Discussion

In this cohort of patients with acute alcohol withdrawal who were discharged from the ED, those who received phenobarbital were less likely to return to the ED within three days of the index visit when compared to those who received benzodiazepines alone. This difference was not present when examining return visits between three and seven days following the index visit. After multivariate adjustment, odds ratios demonstrated no significant differences. While the baseline characteristics were similar among treatment groups, patients who received phenobarbital were much more aggressively treated than patients who received benzodiazepines alone.

Despite multiple studies demonstrating phenobarbital to be an effective medication for acute alcohol withdrawal, few studies have examined the outpatient efficacy of phenobarbital after administration in the ED [10, 12]. In part, this is likely due to difficulty in coordinating follow-up for these patients, who often have unstable housing, comorbid substance use and mental illness, or other socioeconomic barriers precluding them from reliable follow-up. In a prospective, uncontrolled study of patients with alcohol withdrawal, no patient who received a loading dose of phenobarbital in the ED and was subsequently discharged returned within the following week [12]. However, return visits were only queried at the institution of the index visit, and there was no comparison cohort. In a prospective, randomized trial of phenobarbital versus benzodiazepines for acute alcohol withdrawal in the ED, there was no difference in mean CIWA score at 48-h follow-up, but all patients in the benzodiazepine cohort received chlordiazepoxide at discharge, and only 36% of the phenobarbital cohort returned for follow-up [10].

Given the difficulty of prospectively coordinating follow-up in this population, we used return visits as a surrogate marker for ED resource utilization. Our study suggests that phenobarbital decreases ED resource utilization when compared to benzodiazepines within three days of ED discharge, but this difference appears largely driven by the amount of lorazepam equivalents administered during the ED encounter. Several factors may have contributed to phenobarbital patients receiving higher lorazepam equivalents, despite similar baseline characteristics and markers of alcohol withdrawal severity. Much of the published literature either reports or recommends a starting phenobarbital dose of 260 mg IV for moderate to severe withdrawal, with several studies providing greater than 500 mg of IV phenobarbital [6, 8–10, 12]. In contrast, these same studies use a starting lorazepam dose of 2–4 mg, despite this being only one-fourth of the lorazepam equivalents [13]. Both providers and nurses may be uncomfortable with providing true equivalent dosing, approximately 17 mg of IV lorazepam for 260 mg of IV phenobarbital, a phenomenon that has been described with morphine and dilaudid [14, 15]. The factors contributing to the increased dosing of phenobarbital warrant further study.

Phenobarbital is often used as a second line or adjunctive agent for alcohol withdrawal, in part due to the associated risk for delayed respiratory and cardiac depression [5]. There is also some concern that patients who are loaded with phenobarbital in the ED could go on to use alcohol or benzodiazepines once discharged, resulting in synergistic CNS and respiratory depressive effects. While there were many patients in the current study who returned to the ED for alcohol intoxication, we found no significant differences among treatment groups. Despite the frequency of alcohol consumption in close proximity to phenobarbital administration, there were no significant differences in hospitalizations following a return visit between treatment groups. Furthermore, 95% of patients who received phenobarbital were confirmed to be alive beyond three days of the index ED visit, which was similar to the short-term survival rate of patients who received benzodiazepines alone. In a population that is particularly difficult to engage in follow-up, phenobarbital appears to be safe in patients who are discharged from the ED.

In addition to symptom control, there are several potential benefits to using phenobarbital as a first-line agent for alcohol withdrawal in the ED. Phenobarbital has the longest half-life of commonly used sedatives and could obviate the need for discharge medications, such as benzodiazepines or other sedatives [5]. In addition to the potential for misuse or diversion, the need for ongoing GABA agonist therapy often requires a higher level of outpatient care and may limit the options for ongoing AUD treatment following ED discharge. Furthermore, patients with AUD frequently leave medical detoxification units prematurely, citing poorly controlled withdrawal symptoms [16]. Phenobarbital may provide a therapeutic bridge through the initial days of treatment, thereby facilitating increased engagement in outpatient rehabilitation. Finally, patients with alcohol use disorder are more likely to be frequent utilizers of the ED, which is associated with high costs of acute services [17, 18]. Our results suggest that phenobarbital may reduce ED utilization in the short term. If paired with interventions that address the underlying AUD, such as facilitated referral to community care and medication assisted therapy, phenobarbital could potentially deliver cost-savings by preventing repeat ED visits.

This study has several limitations. First, this is a retrospective study, and the groups may be unbalanced to due to lack of randomization. It is possible that the choice of treatment was guided by the severity of withdrawal, which would result in confounding by indication. While we adjusted for multiple covariates in the model, there are certainly residual and unmeasured confounders. Therefore, we cannot establish causal relationships between our outcomes and the received medication. A prospective, randomized trial is needed to confirm these results. Second, alcohol withdrawal is predominately a clinical diagnosis, and thus it is possible that patients without the condition were misdiagnosed and included in the cohort. Third, we did not examine associated discharge prescriptions or undocumented outpatient medication use, which may have impacted return ED visits. However, it is difficult to confirm if a discharge medication is filled or taken appropriately, even if prescribed. Finally, patients may have presented to EDs outside the EHR catchment area, but we sought to minimize this by using a regional health information exchange. Regardless, patients with alcohol use disorder may be transient, accounting for the number of unknown patient outcomes in the cohort.

## Conclusions

Patients with acute alcohol withdrawal who were discharged from the ED after receiving phenobarbital were less likely to return to the ED within three days when compared to patients who received benzodiazepines alone. Despite similar baseline characteristics, patients who received phenobarbital were provided higher lorazepam equivalents. A prospective, randomized trial is needed to verify these results.

## Supplementary Information

Below is the link to the electronic supplementary material.

Supplementary file1 (DOCX 103 KB)
