# Supplementary material for: Return Encounters in Emergency Department Patients Treated with Phenobarbital Versus Benzodiazepines for Alcohol Withdrawal
Source: J Med Toxicol. 2021 Oct 25;18(1):4–10. doi: 10.1007/s13181-021-00863-2 (PMC8758850; doi:10.1007/s13181-021-00863-2)
Supplement: Supplementary file 1 — Supplementary file1 (DOCX 103 KB) [file 13181_2021_863_MOESM1_ESM.docx]

| Supplemental table 1: Association of treatment with phenobarbital, with or without benzodiazepines, vs benzodiazepines alone with repeat ED visits | | | |
| --- | --- | --- | --- |
| **Variable** | **Benzodiazepines without Phenobarbital (n=235)** | **Phenobarbital (n=235)** | **p-value** |
| **ED visit within 3 days, n (%)** | 58 (24.7) | 27 (11.5) | <0.001 |
| **ED visit within 7 days, n (%)** | 76 (32.3) | 44 (18.7) | 0.001 |
| **ED visit 3-7 days after index visit, n (%)** | 18 (10.2) | 17 (8.2) | 0.5 |
|  |  |  |  |
| **Unadjusted Mixed Effects model** |  |  |  |
| **ED visit within 3 days, OR (95%CI)** | Ref | 0.42 (0.24, 0.75) | 0.003 |
| **ED visit within 7 days, OR (95%CI)** | Ref | 0.52 (0.3, 0.91) | 0.03 |
| **ED visit 3-7 days, OR (95%CI)** | Ref | 0.8 (0.36, 1.76) | 0.6 |
|  |  |  |  |
| **Adjusted Mixed Effects model** |  |  |  |
| **ED visit within 3 days, aOR (95%CI)** | Ref | 0.4 (0.22, 0.7) | 0.002 |
| **ED visit within 7 days, aOR (95%CI)** | Ref | 0.48 (0.27, 0.83) | 0.008 |
| **ED visit 3-7 days, aOR (95%CI)** | Ref | 0.73 (0.33, 1.61) | 0.4 |

Supplemental table 2: Frequency of different benzodiazepines used to treat alcohol withdrawal

| Supplemental table: Phenobarbital monotherapy vs Chlordiazepoxide with and without other benzodiazepine | | | |
| --- | --- | --- | --- |
|  | **Chlordiazepoxide (N=165)** | **Phenobarbital Only (N=133)** | **p-value** |
| **ED visit within 3 days, aOR (95%CI)** | Ref | 0.48 (0.23, 0.99) | 0.049 |
| **ED visit within 7 days, aOR (95%CI)** | Ref | 0.48(0.24,0.96) | 0.037 |
| **ED visit 3-7 days, aOR (95%CI)** | Ref | 0.78 (0.22, 2.7) | 0.7, 0.8 |

ICD-10 Codes for Historical Data:

Liver Disease: I86.4, I85.0, I85.9, I98.2, I98.3, K70.0, K70.2, K70.3, K71.7, K74.6, K73.0

Substance use: F11.10, F11.20, F18.10, F19.20, F13.20, F15.20, F15.21

Delirium Tremens: F10.231
